# A novel cell-based screening assay for small-molecule MYB inhibitors identifies podophyllotoxins teniposide and etoposide as inhibitors of MYB activity

**DOI:** 10.1038/s41598-018-31620-1

**Published:** 2018-09-03

**Authors:** Maria Yusenko, Anke Jakobs, Karl-Heinz Klempnauer

**Affiliations:** Institute for Biochemistry, Westfälische-Wilhelms-Universität, D-48149 Münster, Germany

## Abstract

The transcription factor MYB plays key roles in hematopoietic cells and has been implicated the development of leukemia. MYB has therefore emerged as an attractive target for drug development. Recent work has suggested that targeting MYB by small-molecule inhibitors is feasible and that inhibition of MYB has potential as a therapeutic approach against acute myeloid leukemia. To facilitate the identification of small-molecule MYB inhibitors we have re-designed and improved a previously established cell-based screening assay and have employed it to screen a natural product library for potential inhibitors. Our work shows that teniposide and etoposide, chemotherapeutic agents causing DNA-damage by inhibiting topoisomerase II, potently inhibit MYB activity and induce degradation of MYB in AML cell lines. MYB inhibition is suppressed by caffeine, suggesting that MYB is inhibited indirectly via DNA-damage signalling. Importantly, ectopic expression of an activated version of MYB in pro-myelocytic NB4 cells diminished the anti-proliferative effects of teniposide, suggesting that podophyllotoxins disrupt the proliferation of leukemia cells not simply by inducing general DNA-damage but that their anti-proliferative effects are boosted by inhibition of MYB. Teniposide and etoposide therefore act like double-edged swords that might be particularly effective to inhibit tumor cells with deregulated MYB.

## Introduction

Myb proteins constitute is a highly conserved family of transcription factors that are involved in the control of proliferation and differentiation of various cell types^1,2^. MYB, the founding member of the family, was first identified several decades ago as the cellular counterpart of the retroviral protein v-MYB encoded by the oncogene of avian myeloblastosis virus^3–5^. A large body of evidence has shown that MYB is highly expressed in the immature cells of the hematopoietic system and is crucial for the development and homeostasis of the hematopoietic system^1^. MYB is now beginning to attract attention as a potential drug target because recent work has shed light on its relevance for human cancer^6,7^. Genomic rearrangements of the human *MYB* gene and mutations that create de-novo MYB binding sites in transcriptional control regions of the *TAL1* and *LMO1* oncogenes have been detected in acute lymphoid leukemia, suggesting that MYB plays causal roles in the development of these leukemias^8–10^. Importantly, although MYB rearrangements are not detected in the majority of acute myeloid leukemia (AML) cells, these cells are more vulnerable to MYB inhibition than their normal counterparts indicating that they are addicted to high levels of MYB activity^11–13^. Gene rearrangements and deregulation of MYB expression have also been implicated in certain non-hematopoietic tumors, such as breast and colon cancer^14–17^, adenoid cystic carcinoma^18^ and diffuse low-grade pediatric gliomas^19^. Overall, these findings have greatly stimulated interest in MYB as a potential drug target.

The activity of MYB as a transcription factor is highly dependent on its association with the coactivator p300. Attempts to inhibit Myb activity have therefore been focused on the Myb/p300 interaction which is mediated by a highly conserved LXXLL-motif located in the MYB transactivation domain and binds to the KIX-domain of p300^20^. Several studies have firmly established the relevance of the LXXLL motif for MYB activity^21–23^. For example, amino acid substitutions within the LXXLL motif (such as replacement of Leu-302 by Ala) disturb the ability of human AML oncogenes to induce AML. Our own group has recently identified the first low molecular weight compounds that inhibit MYB activity by disrupting the Myb/p300 interaction, thereby providing proof-of-principle that MYB can be targeted effectively by small-molecule inhibitors^7,24–26^.

To identify compounds that inhibit MYB activity we have previously established a reporter cell line based on a GFP reporter gene driven by the cis-elements of the MYB-inducible chicken *MIM1* gene^27^. We noted that some compounds initially identified as potential MYB inhibitors with these cells inhibit the activity of C/EBPβ, a transcription factor cooperating with MYB at the *MIM1* gene^28–30^. To be able to search for MYB inhibitors in a more focused manner we have re-designed the MYB reporter cell line and used it to screen a library of natural compounds. Unexpectedly, this work showed that the topoisomerase II inhibitors teniposide and etoposide also affect MYB activity and its expression in myeloid leukemia cells. This finding suggests that these widely used chemotherapeutic agents have a dual mode of action and might be particularly effective for the treatment of MYB deregulation-dependent tumors.

## Results

### Designing a cell-based screening system for inhibitors of human MYB

**Figure 1:**
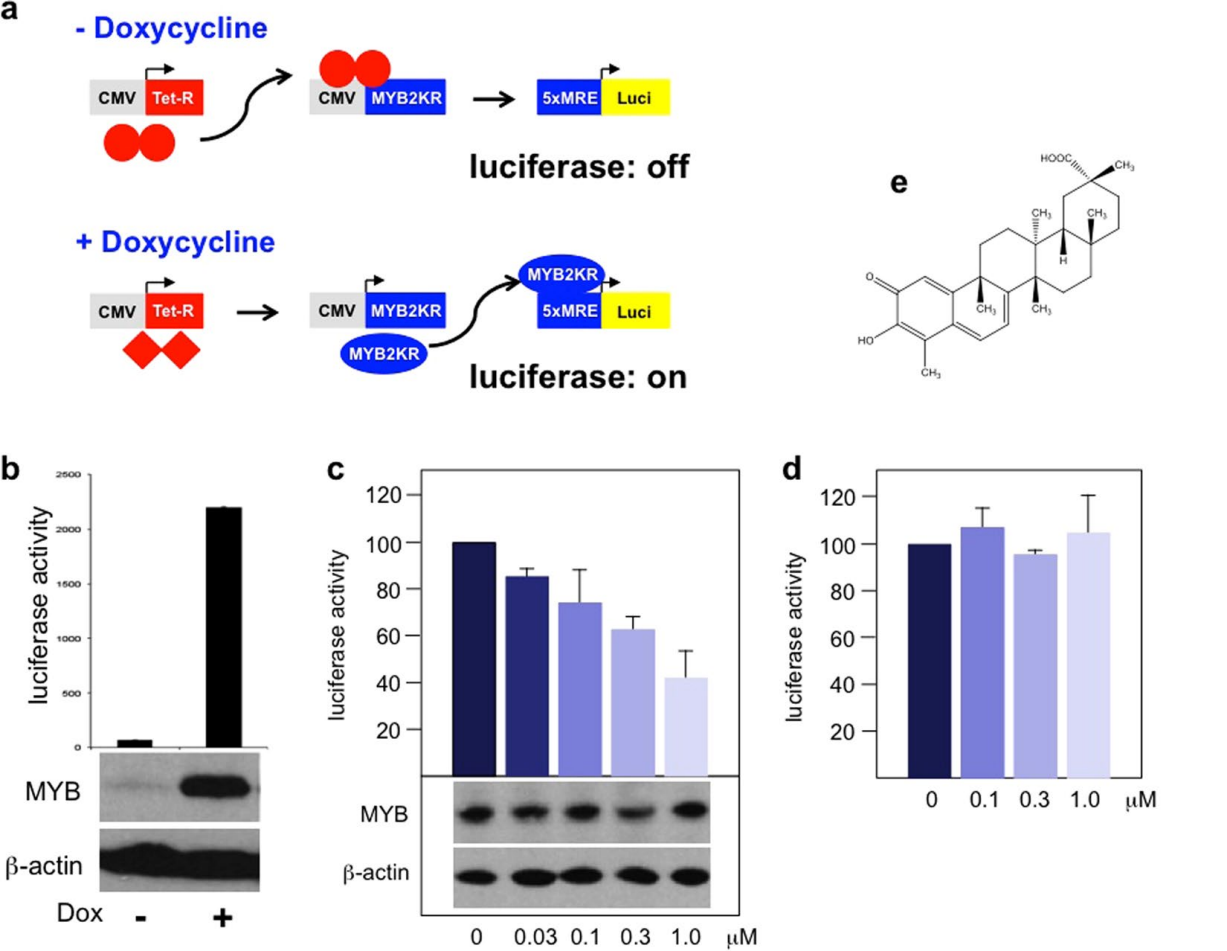
Development of a luciferase-based MYB reporter cell line. (**a**) Schematic illustration of the MYB reporter cell line Hek-Myb-Luc. The cells carry a stably transfected artificial MYB-inducible reporter gene, an expression vector for human MYB(2KR) under the control of a modified CMV promoter with Tet-operator sites close to the transcriptional start site and an expression vector for the Tet-repressor. (**b**) Hek-Myb-Luc cells were grown for 12 hours in the absence or presence of doxycycline. Columns show the mean luciferase activity in arbitrary units with standard deviations. The bottom panels show MYB and β-actin expression in the absence or presence of doxycycline. (**c**) Hek-Myb-Luc cells were treated with doxycycline and Celastrol at the indicated concentrations for 12 hours. Cell extracts were then analyzed for luciferase activity and the expression of MYB and β-actin. (**d**) Luciferase activity of Hek-Luc cells constitutively expressing luciferase treated for 12 hours with the indicated concentrations of Celastrol. Columns and standard deviations in c and d are based on three independent experiments with two replicate samples in each case. (**e**) Structure of Celastrol.

We have previously described an assay for small molecule MYB inhibitors that was based on the myeloid chicken cell line HD11 engineered to express chicken MYB in a doxycycline-inducible manner and to carry a MYB-inducible GFP-reporter gene driven by the promoter and enhancer of the MYB-inducible chicken *MIM1* gene^27^. The identification and characterization of inhibitory compounds with this cell line has shown that candidate inhibitors affect MYB activity also indirectly by targeting the transcription factor C/EBPβ, a crucial co-operation partner required by MYB to induce *MIM1* expression^28–31^. Although small-molecule inhibitors of C/EBPβ are also of interest given that C/EBPβ has pro-oncogenic roles in several tumors^32–36^, we wanted to be able to search for MYB inhibitors in a more focused manner. We therefore generated the reporter cell line illustrated schematically in Fig. 1a. We equipped Hek293 cells with a doxycycline-inducible expression system for a previously described sumoylation-deficient mutant of human MYB (referred to as MYB2KR) that has a higher transactivation potential than the wild-type protein^37,38^. In addition, a luciferase reporter gene driven by a basal promoter and 5 copies of a high-affinity MYB binding site was stably introduced into the cells. Figure 1b shows that the luciferase activity was strongly induced when the cells were grown in the presence of doxycycline. Furthermore, western blotting confirmed the induction of MYB expression by doxycycline.

To test the performance of the cell system (referred to as Hek-Myb-Luc), we treated the cells with Celastrol, a previously identified MYB inhibitor^25^. As shown in Fig. 1c, addition of Celastrol diminished the luciferase activity in a dose-dependent manner. The amount of MYB was not decreased by Celastrol, confirming that Celastrol inhibits the activity and not the expression of MYB. We also generated a companion cell line (referred to as Hek-Luc) by introducing a CMV promoter-driven constitutively active luciferase expression vector into Hek293 cells. When these cells were treated with Celastrol, no decrease of luciferase activity was observed, indicating that Celastrol does not inhibit the activity of the luciferase itself (Fig. 1d).

### Screening of a natural compound library

**Figure 2:**
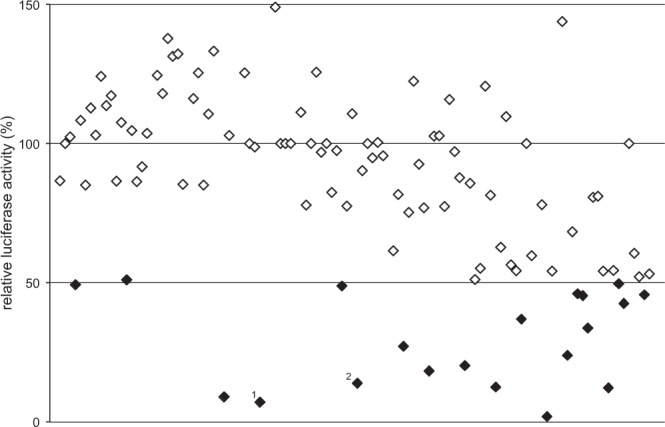
Screening the “Natural product set III” with Hek-Myb-Luc cells. Each compound of the library is represented by a dot. The y-axis shows the luciferase activity after treating Hek-Myb-Luc cells for 12 hours with 5 μM of each compound. Dots for compounds with 50% inhibition or less are shown in black. Numbers refer to toyocamycin^1^ and teniposide^2^.

**Figure 3:**
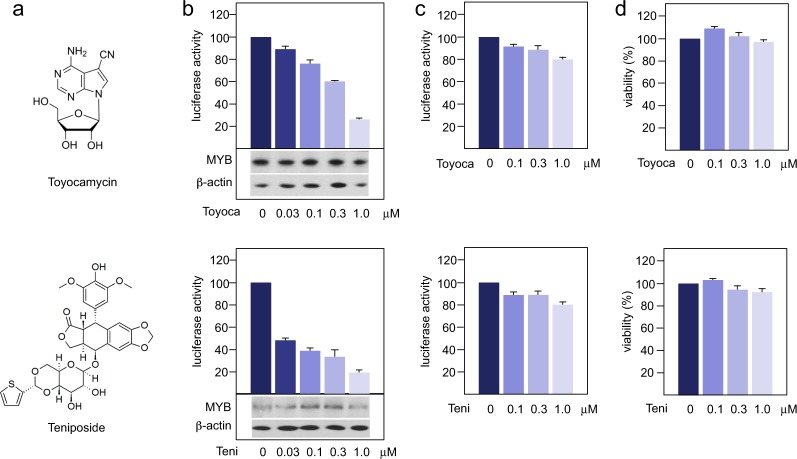
Myb-inhibitory activity of selected compounds. The figure shows the chemical structures of toyocamycin and teniposide (**a**), the luciferase activity of Hek-Myb-Luc cells (**b**) or Hek-Luc cells (**c**) after treatment for 12 hours with the respective compound and the impact of each compound on the viability of the Hek-Myb-Luc cells (**d**). The bottom panels in b show the expression of MYB and β-actin. Columns and standard deviations for luciferase activity are based on three independent experiments with two replicate samples in each case. Viability assays are based on two independent experiments with six replicate samples in each case.

We used the Hek-Myb-Luc cells to screen the “Natural products set III”, a collection of approximately 120 natural compounds obtained from the National Cancer Institute (http://www.dtp.nci.nih.gov). Figure 2 and Supplementary Table 1 provide an overview of the inhibitory activities of the compounds at 5 μM concentration. Compounds, which showed inhibitory activity or cell toxicity were tested again using lower concentrations. These compounds were also tested for inhibitory activity towards luciferase using the Hek-Luc cells. This resulted in the final identification of two compounds, toyocamycin and teniposide, which significantly inhibited luciferase activity in the MYB-dependent cell line while showing only slight effects on luciferase activity in the control cells and on cell viability (Fig. 3). Especially in the case of teniposide significant inhibition of MYB activity was observed at nanomolar concentrations while MYB expression itself was not affected, indicating that the compounds inhibit the activity and not the expression of MYB. Overall, our data demonstrate that the re-designed screening system described here can be used successfully to identify compounds that inhibit MYB activity.

### Teniposide induces myeloid differentiation of NB4 cells in a MYB-dependent manner

Teniposide is a known chemotherapeutic agent that acts as a topoisomerase II inhibitor^39^ and has been used for the therapy of certain tumors, such as childhood acute lymphocytic leukemia. We were intrigued by its ability to inhibit MYB activity as this suggested a dual mode of action that might be particularly effective for the therapy of tumors with de-regulated MYB. This prompted us to investigate the MYB inhibitory activity of teniposide in more detail. Since the screening assay was based on the inhibition of the MYB-2KR mutant we first investigated if teniposide also inhibits the activity of wt MYB. A comparison of the effect of teniposide on Myb-wt and Myb-2KR showed that the activity of both proteins was inhibited, however, the wt protein was inhibited slightly less efficiently than the mutant protein (Supplementary Fig. 1).

**Figure 4:**
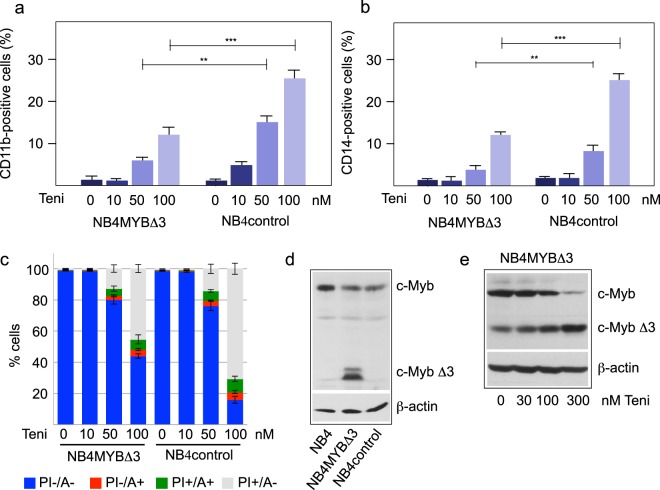
Analysis of teniposide-induced differentiation and cell death of NB4 cells. (**a**,**b**) NB4 cells expressing a truncated MYB protein (NB4Δ3) and NB4 control cells were treated for two days with the indicated concentrations of teniposide followed by staining with antibodies against CD11b (**a**) or CD14 (**b**). The bars show the percentage of positive cells among the fraction of viable cells, as determined by flow cytometry. (**p < 0.01; ***p < 0.001, Student’s t-test). (**c**) The cells were treated as in a,b followed by staining with annexin V and propidum iodide. Fractions of cells positive or negative for staining with annexin V or propidium iodide are indicated by bar graphs. (**d**) Western blot of total extracts of wild-type NB4 cells, NB4 cells infected with a lentivirus encoding MYBΔ3 and NB4 cells infected with a control lentivirus with antibodies against MYB and β-actin. (**e**) Western blot of total extracts of NB4MYBΔ3 cells treated for 12 hours with the indicated concentrations of teniposide. The blot was developed with antibodies against MYB and β-actin.

Previous work has shown that down-regulation of MYB expression in immature hematopoietic cells induces their differentiation and/or cell death^1^. We used the promyelocytic leukemia cell line NB4 to study the effect of teniposide on differentiation and survival of the cells. Importantly, we also employed NB4 cells expressing a C-terminally truncated version of MYB (referred to as MYBΔ3) that is more active than the full-length protein^40^ and can be distinguished by western blotting from the wild-type protein, as shown in Fig. 4d. We reasoned that the expression of this activated version of MYB would allow us to distinguish MYB-dependent from MYB-independent effects of the compound. To see if teniposide induces differentiation we treated the cells for two days with the compound, thus allowing accumulation of a significant numbers of differentiated cells. Figure 4a,b shows that teniposide induced wild-type NB4 cells to differentiate in a concentration-dependent manner, as demonstrated by flow cytometry using the differentiation markers CD11b and CD14. Interestingly, MYBΔ3 expressing cells differentiated less in the presence of teniposide compared to the control cells, suggesting that teniposide induces differentiation, at least in part, by inhibition of MYB. As shown in Fig. 4c, teniposide also induced cell death, as determined by annexin V and propidium iodide staining of the cells. Importantly, at high concentration of teniposide the MYBΔ3-expressing cells showed less cytotoxic effects, again suggesting that teniposide causes these effects in part by the inhibition of MYB. Supplementary Fig. 1 confirms that the truncated MYB protein is also strongly inhibited by teniposide. Interestingly, analysis of the expression of full-length and truncated MYB showed that the expression of full-length MYB was decreased in the presence of teniposide (Fig. 4e). By contrast, the expression of the C-terminally truncated MYBΔ3 was not decreased under these conditions, providing a possible explanation for its ability to partially counteract the cytotoxic effect of teniposide.

### Teniposide down-regulates MYB expression in AML cell lines

**Figure 5:**
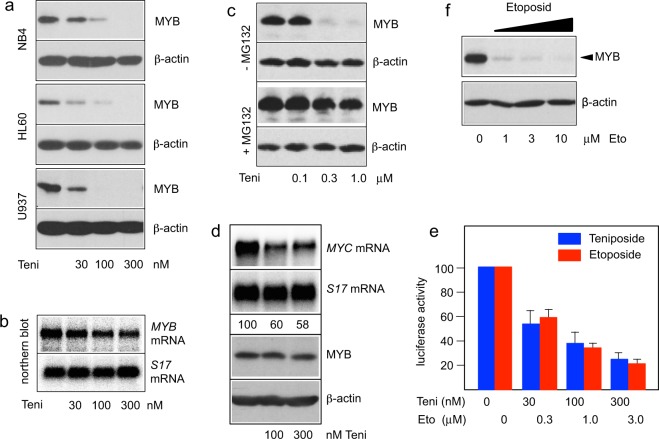
Teniposide down-regulates MYB expression in myeloid leukemia cell lines. (**a**) NB4, HL60 and U937 human leukemia cells were treated for 24 hours with teniposide at the indicated concentrations. Total cell extracts were then analyzed by western blotting for MYB and β-actin expression. (**b**) mRNA isolated from HL60 cells treated for 24 hours with different concentrations of teniposide was analyzed by northern blotting for expression of MYB and ribosomal protein S17 mRNAs. (**c**) HL60 cells were treated for 5 hours with the indicated concentrations of teniposide in the absence or presence of the proteasome inhibitor MG132, followed by western blot analysis of MYB and β-actin expression. (**d**) NB4 cells were pre-treated for 30 min with MG132 and then incubated for additional 4 hours with the indicated concentrations of teniposide. Polyadenylated RNA isolated from the cells was then analyzed by northern blotting for expression of *MYC* and *S17* mRNAs. Numbers below the lanes indicate the amounts of *MYC* mRNA relative to *S17* mRNA, determined by quantification with a phosphor image analyzer. Western blot analysis of MYB and β-actin expression in total cell extracts is shown below. (**e**) Luciferase activity of Hek-Myb-Luc cells treated for 12 hours with doxycycline and the indicated concentrations of teniposide and etoposide. Bars show luciferase activities with standard deviations and are based on three independent experiments with two replicate samples in each case. (**f**) HL60 cells treated for 24 hours with different concentrations of etoposide were analyzed as in a.

To further characterize the effect of teniposide on myeloid cells, we performed western blot analyses of teniposide-treated NB4 cells. Interestingly, teniposide induced a strong concentration-dependent decrease of the MYB expression level (Fig. 5a). Similar observations were made for HL60 and U937 cells, indicating that down-regulation of MYB expression by teniposide is common to several myeloid leukemia cell lines. To understand how teniposide down-regulates MYB expression we examined if teniposide decreases the amount of MYB mRNA. We treated HL60 cells for 16 hours with and without teniposide and analyzed the expression of MYB mRNA by northern blotting. However, we observed only a mild decrease of the MYB mRNA level that could not account for the almost complete absence of MYB at the highest concentration of teniposide (Fig. 5b). It therefore appeared that the down-regulation of MYB by teniposide occurs post-transcriptionally, presumably by degradation of the protein. To confirm this we examined the effect of the proteasome inhibitor MG132 on the teniposide-induced decrease of MYB expression. To minimize toxicity of MG132 we treated HL60 cells only for 5 hours with teniposide in the absence or presence of MG132. Western blotting showed that somewhat higher concentrations of teniposide were needed to observe a strong down-regulation of MYB expression compared to overnight treatment of the cells. Importantly, MG132 prevented the down-regulation of MYB expression, indicating that teniposide induces proteasomal degradation of MYB (Fig. 5c).

The fact that teniposide inhibited MYB activity without inducing its degradation in the Hek-Myb-Luc cells whereas MYB expression was decreased by teniposide in different AML cell lines raised the question of whether teniposide acts in completely different manner in Hek cells versus myeloid cells. In order to demonstrate that teniposide also inhibits MYB activity in AML cells we treated NB4 cells only for 4 hours with teniposide in the presence of MG132. Under these conditions we expected MYB not to be degraded. We then examined the expression of the endogenous *MYC* gene, a well-known MYB target gene, whose mRNA has an extremely short half-life^41^. Figure 5d shows that the expression of *MYC* mRNA significantly decreased in the presence of teniposide while the amount of MYB remained stable, indicating that teniposide inhibits MYB activity also in myeloid cells and suggesting that induction of degradation of MYB is an additional effect of teniposide in myeloid cells.

We also investigated whether the related Topoisomerase II inhibitor etoposide has similar effects on MYB as teniposide. Comparison of the inhibitory activities of both compounds showed that etoposide also inhibits MYB activity in Hek-Myb-Luc cells but that approximately 10 times higher concentrations of etoposide than of teniposide were required to achieve similar inhibition (Fig. 5e). This is consistent with earlier studies that showed that the cytotoxic activity of teniposide in various tumor cells was approximately 10 times higher than that of etoposide^42,43^. Etoposide also showed a similar reduction of MYB expression in HL60 cells, except that a 10-fold higher concentration was again required to achieve a similar effect (Fig. 5f).

### Caffeine partially rescues MYB activity in the presence of teniposide

**Figure 6:**
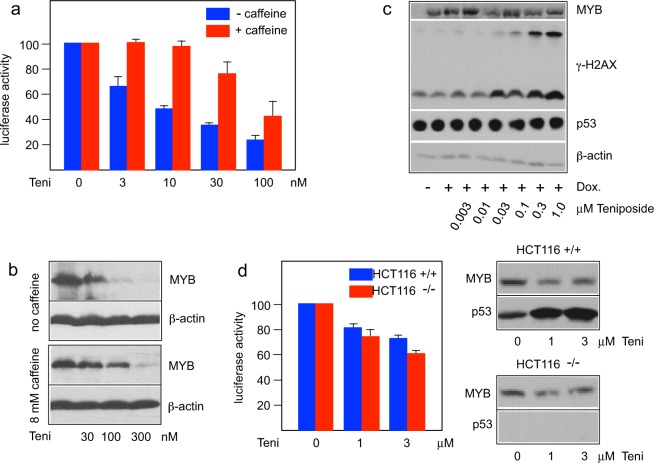
Inhibition of MYB activity by teniposide is mediated by DNA-damage signaling. (**a**) Hek-Myb-Luc cells treated for 12 hours with doxycycline, different concentrations of teniposide and without (blue bars) or with 8 mM caffeine (red bars). Columns and standard deviations are based on four independent experiments with two replicate samples in each case. (**b**) HL60 cells were treated for 24 hours with different concentrations of teniposide in the absence or presence of caffeine. Total cell extracts were then analyzed by western blotting for MYB and β-actin expression. (**c**) Western blot analysis of MYB, γ-H2AX, p53 and β-actin expression of Hek-Myb-Luc cells treated as in a. The slower migrating band in the γ-H2AX panel corresponds to ubiquitinated γ-H2AX. (**d**) HCT116 wt and p53 knock-out cells were transfected with the MYB-inducible luciferase reporter gene pGL4-5xMRE(GG)-Myc, the constitutive β-galactosidase expression vector pCMVβ and expression vector for MYB(2KR). The columns (left panel) show the luciferase activity, normalized against β-galactosidase activity, of the cells after treatment for 12 hours with the indicated concentrations of teniposide. Standard deviations are based on three independent experiments with two replicate samples in each case. Western blot analysis (right panels) of MYB and p53 expression in HCT116 wt and p53 knock-out cells.

Inhibition of topoisomerase II induces various DNA-damage, such as DNA double strand breaks (DSBs), and thereby activates the DNA-damage response to orchestrate the DNA repair and other downstream events. We therefore examined if the inhibition of MYB activity by teniposide is mediated by DNA-damage signaling. To do this, we treated the MYB reporter cell line with teniposide in the absence or presence of caffeine, a compound that is known to inhibit DNA-damage signaling mediated by the protein kinases ATM and ATR^44^. Interestingly, Fig. 6a shows that caffeine rescued MYB activity, particularly at the lower concentrations of teniposide whereas the rescuing effect was only partial at the higher concentrations. Similarly, the teniposide-induced decrease of MYB in leukemia cells was also retarded in the presence of caffeine. When HL60 cells were treated with teniposide in the absence and presence of caffeine the decrease of MYB was less pronounced in the presence of caffeine (Fig. 6b). This was especially obvious at the lower concentrations of teniposide where a concentration of 100 nM teniposide resulted in an almost complete loss of MYB in the absence of caffeine whereas MYB levels were only slightly decreased when caffeine was present. Together these findings suggested that inhibitory effects of teniposide were caused indirectly, at least at lower concentrations, by the activation of a DNA-damage signaling pathway.

To corroborate the notion that MYB activity is modulated in response to DNA-damage we tested the effects of two additional DNA-damage inducing compounds, 9-amino-camptothecin and the anthracyclin mitoxantrone, that are structurally unrelated to the podophyllotoxins teniposide and etoposide. Camptothecin induces DNA damage by inhibiting topoisomerase I^45^, and mitoxantrone is a topoisomerase II poison^46^. As shown in Supplementary Fig. 2 both compounds reduced the MYB transactivation potential in the Hek-Myb-Luc cells similar to teniposide.

A previous report has claimed that the tumor suppressor protein p53 inhibits MYB-dependent transactivation by direct binding to the MYB DNA-binding domain^47^. To address if the inhibitory effects elicited by teniposide might be due to increased p53 expression we analyzed the teniposide-treated cells by western blotting (Fig. 6c). This confirmed that teniposide leads to a concentration-dependent increase of γ-H2AX, a marker of DBSs, but there was no obvious increase of the expression of p53 under these conditions. To further address the role of p53 in the teniposide-dependent Myb expression we compared the ability of teniposide to inhibit Myb activity in HCT116 wild-type and p53 knock-out cells. As illustrated in Fig. 6d, teniposide had similar effects on MYB in both cell-lines, which also argues against an important role of p53 in the inhibitory mechanism. We noted that the concentrations of teniposide required for MYB inhibition were significantly higher in these cells than in the Hek293-derived reporter cell line. This difference might be a cell-line specific effect or related to the transient versus stable introduction of the MYB expression vector and luciferase reporter constructs in the two cell systems. Finally, the observation that teniposide causes down-regulation of MYB expression in HL60 cells, which are negative for p53 expression^48^, further indicates that the teniposide-induced decrease of MYB expression is independent of p53.

## Discussion

Recent progress in understanding the role of MYB in the development human leukemia and other tumors has made MYB an attractive target for the development of small-molecule inhibitors. Several recent reports have demonstrated that MYB is a druggable target whose inhibition might permit new therapeutic strategies for the treatment of AML^25,26,49–51^. To facilitate the identification of such compounds we have developed a reporter cell line that is based on the doxycycline-inducible expression of an activated version of human MYB acting upon a stably integrated MYB-dependent luciferase reporter. This reporter combines a minimal promoter with a cluster of high-affinity MYB binding sites leading to highly MYB-dependent luciferase expression, as demonstrated by the high fold-induction of luciferase activity by MYB. We have previously established a reporter cell line that is based on a GFP-reporter gene driven by a complex natural promoter and enhancer of a natural MYB-regulated gene^27^. By contrast, the cell system described here is more focused on detecting inhibitors of Myb itself rather than of other proteins that cooperate with MYB at the promoter and enhancer of a natural MYB target gene. To aid the identification of “false-positive” compounds we have generated a second cell line that constitutively expresses luciferase from the CMV promoter and can be used to quickly check candidate compounds for unspecific inhibition of the luciferase itself.

To test the performance of the cell system we have screened a library of natural products and identified two compounds - toyocamycin and teniposide - that inhibit the stimulation of the reporter gene by MYB at sub-micromolar concentrations but have only a little effect on the luciferase itself. Toyocamycin is a nucleoside antibiotic from the gram-positive bacterium *Streptomyces toyocaensis* that has been studied for its cytotoxic effects on several cancer cell lines and reported to inhibit RNA synthesis^52,53^ and the activity of certain kinases^54,55^. Furthermore, toyocamycin has been shown to target the ER-stress response in multiple myeloma and pancreatic cancer cells^56,57^. Teniposide is a podophyllotoxin derivative acting as a potent topoisomerase II inhibitor. Teniposide and the related compound etoposide are well-known chemotherapeutic agents employed in the therapy of leukemia and solid tumors, usually in combination with other drugs. Overall, the identification of potent inhibitory compounds demonstrates that the MYB reporter cell line described here will be a useful screening tool for compounds that inhibit MYB. Screening of a large compound library with this cell line is currently under way.

The fact that the podophyllotoxin derivatives teniposide and etoposide are used in the chemotherapy of patients with leukemia and other cancers has prompted us to study the teniposide-induced inhibition of MYB in more detail. Teniposide and etoposide are potent cytotoxic agents that inhibit topoisomerase II to cause DNA-damage in proliferating cells. It was surprising that these compounds also have profound inhibitory effects on MYB activity and expression. In the Hek-Myb-Luc reporter cell line teniposide inhibited Myb activity already at concentrations as low as 3 nM (Fig. 6a) but did not induce an obvious decrease of the MYB expression level. By contrast, we observed a strong reduction of MYB expression in several myeloid leukemia cell lines that was apparently due to the degradation of the protein.

Analysis of the expression of the *MYC* gene, a *bona fide* MYB target gene, confirmed that under conditions where degradation of MYB was prevented by the proteasome inhibitor MG132 (Fig. 5d), MYB activity was also inhibited by teniposide in myeloid cells. It therefore appears that teniposide in myeloid cells acts on MYB in two different ways, namely by inhibiting its activity and by inducing its degradation. How MYB activity is inhibited by teniposide and whether degradation of MYB is causally related to the inhibition of its activity is currently not known and remains to be investigated in future work. A key role of MYB in the hematopoietic system is to maintain an immature phenotype of hematopoietic progenitor cells. Using NB4 cells we have indeed demonstrated that teniposide induces the expression of differentiation markers CD11b and CD14. Interestingly, this was reversed significantly by the expression of an activated version of MYB. Teniposide also induced prominent cytotoxic effects in NB4 cells, especially at higher concentration of the compound, which were also partially rescued by the truncated version of MYB. These findings are interesting because they suggest that the anti-proliferative effects of the podophyllotoxins in myeloid leukemia cells are in part due to the inhibition of MYB. This, in turn, raises the intriguing possibility that these compounds might be particularly effective as chemotherapeutic agents for the treatment of tumors that are driven by deregulated MYB, like AML or childhood T-ALL, where addiction to high levels of MYB expression or *MYB* rearrangements and de-novo MYB binding sites upstream of the *TAL1* gene have been reported in a number of cases^8–13^.

The inhibitory effect of teniposide on MYB activity was partially rescued by caffeine, suggesting that the inhibition is in part dependent on signaling pathways induced by DNA-damage. This is also supported by the observation that DNA-damaging agents structurally unrelated to teniposide had a similar inhibitory effect on MYB activity, suggesting that MYB is a downstream target in the DNA-damage response. A previous report has shown that overexpression of p53 decreases MYB activity by binding to MYB and recruiting the co-repressor mSin3A^47^. However, our data show that teniposide inhibits MYB activity also in the absence of p53, arguing against a direct role of p53 in the inhibitory mechanism of teniposide. As mentioned before, teniposide induced a decrease of MYB expression in several myeloid leukemia cell lines, which appears to be mainly due to proteasomal degradation of MYB rather than altered transcription or RNA stability. This was confirmed by the finding that the proteasome inhibitor MG132 counteracted the inhibition of MYB expression by teniposide while the MYB mRNA level was only slightly affected by teniposide. The decrease in MYB expression by teniposide was also partially rescued by caffeine, consistent with MYB being a downstream target of DNA-damage signaling also in myeloid cells. The fact that HL60 cells are p53-negative^48^ further supports the notion that Myb down-regulation is p53-independent. Myb is a relatively short-lived protein with a half-life of approximately 30 min to 1 hour^58,59^, however the mechanism of its degradation is not yet fully understood. Recent work has implicated the F-box protein Fbw7 in controlling ubiquitin-dependent degradation of MYB^60,61^. Fbw7 acts as a substrate-specific adapter of the SCF-Fbw7 E3 ubiquitin ligase complex. Interestingly, the adenovirus E1A protein interacts with the SCF-Fbw7 complex and inhibits its activity^62^. Since the Hek-Myb-Luc cells express E1A, this might also explain why MYB is not degraded in these cells. The SCF-Fbw7 complex has been implicated in the DNA damage-induced degradation of several proteins, including MYC^63^ and SOX9^64^ among others. It will be interesting to address if MYB is degraded in teniposide-treated myeloid cells in a similar manner, however, this will require further work.

In summary, we have established a highly MYB-dependent reporter cell line that will be useful to identify MYB inhibitory compounds. By using these cells our work has revealed that the anti-proliferative effects of the chemotherapeutic drugs teniposide and etoposide in leukemic cells are in part due to the inhibition of MYB.

## Methods

### Cells and compounds

NB4, HL60 and U937 are human myeloid leukemia cell lines. Hek293 is a human embryonic kidney cell line. HCT116 wild-type and p53-negative cells are human colon carcinoma cell lines. The “Natural products set III” was obtained from the National Cancer Institute (http://www.dtp.nci.nih.gov). Teniposide, etoposide, 9-amino-camptothecin, mitoxantrone and caffeine were obtained from Sigma and stored as 10 mM stock solutions (in DMSO) at −70 °C.

### Construction of the Hek-Myb-Luc and Hek-Luc reporter cell lines

To generate the MYB-reporter cell line a doxycyclin-inducible expression vector for the 2KR mutant of full-length human MYB^37,38^ was first constructed by inserting the MYB2KR coding region into pCDNA4/TO/myc-His-A (Invitrogen). The resulting plasmid was then transfected together with pCDNA6-TR (encoding the Tet-repressor) into Hek293 cells, followed by selection of stable transfectants in the presence of zeocin and blasticidin. Double-resistant cell clones were then analyzed by western blotting for doxycyclin-inducible expression of MYB. A positive clone (Hek293Myb2KR) was subsequently transfected with the luciferase reporter gene pGL4-5xMRE(GG)-Myc (containing 5 tandem copies of a MYB binding site upstream of a core promoter)^38^ and the coding region for luciferase into Hek293Myb2KR cells to finally obtain Hek-Myb-Luc cells. Hek-Luc cells were generated by transfection of a pCDNA3-luciferase construct into Hek293 cells, followed by selection of stable clones in the presence of G-418 and isolation of a positive clone.

### Transfections

Transfection of Hek293 and HCT116 wild-type and p53 knock-out cells by calcium-phosphate co-precipitation and reporter assays were performed as previously described^31^.

### Lentiviral infection

A lentivirus encoding c-terminally truncated MYBΔ3 was generated by replacing the RFP coding region of plasmid pLVX-DsRed-Monomer-C1 (Clontech) with the coding sequence of human MYB truncated after amino acid 390^26^. To generate a control lentiviral vector the RFP coding region was deleted from pLVX-DsRed-Monomer-C1. The lentiviral DNA was transfected together with the packaging plasmids pPAX.2, pMD2.G and pAdvantage (Invitrogen) by calcium-phosphate co-precipitation into Hek293T cells to generate infectious viral particles. NB4 cells (2.5 × 10^4^ cells/well) were then infected with supernatants containing lentivirus with polybrene (8 µg/ml) in 6-well plate pre-coated with RetroNectin at 20 °C at 850 × g for 90 min. Infected cells were selected with 2 μg/ml puromycin until all uninfected NB4 cells died.

### Viability assays

Cell viability was determined by an MTS assay. Cells were incubated with the desired compounds for 48 hours, followed by addition of MTS solution (CellTiter 96_ Aqueous One Solution, Promega) to the medium and incubation for 1 h. The absorbance at 492 nm was measured with a microplate photometer (MPP 4008, Mikrotek).

### Flow cytometry

One million NB4 cells were cultured for 2 days in RPMI 1640 medium containing the desired concentration of teniposide. Control cells were incubated without teniposide. The cells were then stained with anti-human CD11b-FITC (clone ICR F44, BioLegend) or CD14-FITC antibody (clone 63D3, BioLegend) and analyzed by flow cytometry. Side scatter and forward scatter profiles were used to exclude debris and non-viable cells. To analyze the fraction of dead cells, the cells were double-stained with FITC-annexin-V (BioLegend) and propidium iodide (PI) to determine the percentages of apoptotic/necrotic cells.

### Protein and RNA analysis

Total cell extract was prepared by boiling the cells in SDS-sample buffer. Aliquots of the extracts were then analyzed by SDS polyacrylamide gel electrophoresis and western blotting with the following antibodies: Myb (5E11, Sleeman, 1993), β-actin (Sigma-Aldrich, AC-15), p53 (Sigma-Aldrich, DO-1), γ-H2AX (Genetex, GTX61796). Preparation of polyadenylated RNA and northern blotting was performed as described^31^. Northern blots were hybridized sequentially with radiolabeled probes derived from cDNA clones of the respective genes and the intensities of the resulting bands were quantified with a phosphor imager.

## Electronic supplementary material

Supplementary data
