# Supplementary material for: A novel cell-based screening assay for small-molecule MYB inhibitors identifies podophyllotoxins teniposide and etoposide as inhibitors of MYB activity
Source: Sci Rep. 2018 Sep 3;8:13159. doi: 10.1038/s41598-018-31620-1 (PMC6120916; doi:10.1038/s41598-018-31620-1)
Supplement: Supplementary file 1 — Supplementary data [file 41598_2018_31620_MOESM1_ESM.pdf]

Supplementary information

A novel cell-based screening assay for small-molecule MYB inhibitors identifies podophyllotoxins teniposide and etoposide as inhibitors of MYB activity

Maria Yusenko, Anke Jakobs and Karl-Heinz Klempnauer*

Institute for Biochemistry, Westfälische-Wilhelms-Universität, D-48149 Münster, Germany

* Corresponding author: K.-H. Klempnauer, Institute for Biochemistry, Westfälische-Wilhelms-Universität, D-48149 Muenster, Germany. Tel.: +49-251-8333203, Fax: +49-251-8333206, e-mail: klempna@uni-muenster.de

Supplementary Table 1			
	Name	NSC	% Luc
1	Colchicine	757	155
2	(Dimethylamino)ethanol	2652	87
3	Actinomycin D	3053	
4	Pomiferin	5113	102
5	Lambdamycin	5159	49
6	Narcotine	5366	108
7	Veratridine	7524	85
8	Aleuritic acid	7668	113
9	Khellin	8519	103
10	Fumagillol	9665	124
11	Protopine hydrochloride	11440	114
12	Aristolochic acid	11926	117
13	Resorufin	12097	87
14	Chlorotetracycline hydrochloride	13252	108
15	Glaucarubin	14975	51
16	Amygdalin	15780	105
17	Pyrethrosin	22070	86
18	Coumestrol	22842	92
19	Himbacine	23969	104
20	Rotenone	26258	157
21	Cyclophosphamide	26271	124
22	β-Lapachone	26326	118
23	Dihydroisorescinnamine	29854	138
24	Mangostin	30552	131
25	Neohesperidin	31048	132
26	Decarboxy norlobaric acid	31867	85
27	Bicuculline	32192	182
28	Isocorydine	32979	116
29	Curcumin	32982	125
30	Solanine hydrochloride	35611	85
31	Canadine	36351	111
32	(2*R*,3*R*) 2-(3,4-Dihydroxyphenyl)-3,6,7-trihydroxy-2,3- dihydro-4*H*-chromen-4-one	36398	133
33	Santonin oxime	42038	162
34	Streptonigrin	45383	9
35	Methoxsalen	45923	103
36	Carbomycin	51001	161
37	Aconitine	56464	164
38	Fumagillin dicyclohexylamine salt	58368	125
39	Tylocrebrine	60387	
40	Isopropylideneazastreptonigrin	62709	99
41	Toyocamycin	63701	7
42	Vincristine sulfate	67574	305
43	Ellipticine	71795	200
44	Pseudoyohimbine	72116	149
45	Thaspine acetate	76022	
46	Daunomycin hydrochloride	82151	
47	Helenalin	85236	100
48	Parthenicin	85239	238
49	Stictic acid	87511	111

50	Brefeldin A	89671	78
51	Camptothecin	94600	
52	7,8-Dimethylalloxazine	96911	126
53	Fungichromin	105388	97
54	Cucurbitacin E	106399	
55	Ascochitine	114344	82
56	Radicinin	118343	98
57	Valinomycin	122023	49
58	Sporidesmolide I	122224	77
59	Geldanamycin	122750	111
60	Teniposide	122819	14
61	Picrotin	129536	90
62	Tubulosine	131547	100
63	Rifamycin sodium salt	133100	95
64	Lankacidin C	145118	100
65	Nystatin	150817	96
66	Maytansine	153858	161
67	Parthenolide	157035	61
68	Streptoval C	169627	82
69	Fastigillin B	176503	27
70	Lasalocid	177406	75
71	Virginiamycin S_1	177858	122
72	(7*E*)-2-Acetyl-2,3,4,5-tetrahydrooxonine-6,9-dione	180515	93
73	4-(4,5-Dihydroxy-4,6-dimethyloxan-2-yl)oxy-14-ethyl- 6,7,12-trihydroxy-3,5,7,9,11,13-hexamethyl- oxacyclotetradecane-2,10-dione	209870	77
74	Crassin	210236	18
75	Wortmannin	221019	103
76	Rapamycin	226080	103
77	Dodecahydro-8''-hydroxy-1'',6''-dimethyldispiro-[furan- 3(2*H*),2'(5'*H*)-furan-5',5''-[5*H*-1,4a](methan- oxymethano)naphthalen]-2,9''-dione	250429	77
78	(7a*R*,12a*R*) 13-Methoxy-3,3-dimethyl-11-(3-methyl-2- buten-1-yl)-7a,12a-dihydro-3*H*,7*H*-benzofuro[3,2- *c*]pyrano[3,2-*g*][1]benzopyran-10-ol	250430	116
79	17-Amino-17-demethoxygeldanamycin	255109	97
80	Erythro-9-(2-Hydroxy-3-nonyl)adenine hydrochloride	263164	88
81	Nanaomycin A	267461	20
82	Dehydrocyclobutatusin	270914	86
83	Batyl alcohol	284200	51
84	(3a*R*,5aβ,6*S*,8a*S*,8b*S*)-Octahydro-10,10-dimethyl- 6,8b-Ethano-8b*H*-cyclopenta[*de*]-2-benzopyran-1,4- dione	284437	55
85	Siomycin A	285116	121
86	Physalin B	287088	81
87	Nigericin sodium salt	292567	13
88	Daphnetin diacetate	301683	63
89	BOHLMANN K2631	302289	110
90	Cytochalasin H	305222	56
91	Lonchocarpic acid	307981	54
92	Bactobolin	325014	37
93	Didemnin B	325319	
94	Baccatin III	330753	60
95	Rhizoxin	332598	528
96	Hispanolone	332876	78
97	Tetrocarcin A sodium salt	333856	2

98	Triptolide analog	337783	54
99	12-*O*-Palmitoyl-16-hydroxyphorbol-13-acetate	338250	643
100	Bryostatin 1	339555	144
101	Chaetochromin A	345647	24
102	4-Ipomeanol	349438	68
103	Medicarpin	350085	46
104	Sesquiterpene lactone 326	361902	45
105	Artemisinin	369397	34
106	Confertifoline	375294	81
107	Nordracorubin	376248	81
108	Benzyl 4'-(1,3-dioxoisoindol-2-yl)-2,2-dimethyl-1,5'- dioxospiro[3a*H*-imidazo[1,2-a]-indole-4,2'-oxolane]-3- carboxylate	382796	54
109	Illudin M	400978	12
110	Pleurotin	401005	55
111	Ellagic acid	407286	50
112	Corynanthine	407306	43
113	Echinomycin	526417	
114	(1*S*,7*R*,8a*R*)-1,2,3,5,6,7,8,8a-Octahydro-indolizine- 1,6,7,8-tetrol	614552	61
115	Pentoxifyllin	637086	52
116	Michellamine B diacetic acid salt	661755	46
117	Fumitremorgin C	719655	53

Supplementary Table 1. Compounds in the Natural product set III and luciferase activities (percent of untreated cells) after over-night treatment of Hek-Myb-Luc cells at 5 μM concentration. Luciferase values represent the average of two independent measurements. Missing luciferase values refer to cells that appeared dead upon microscopic inspection.

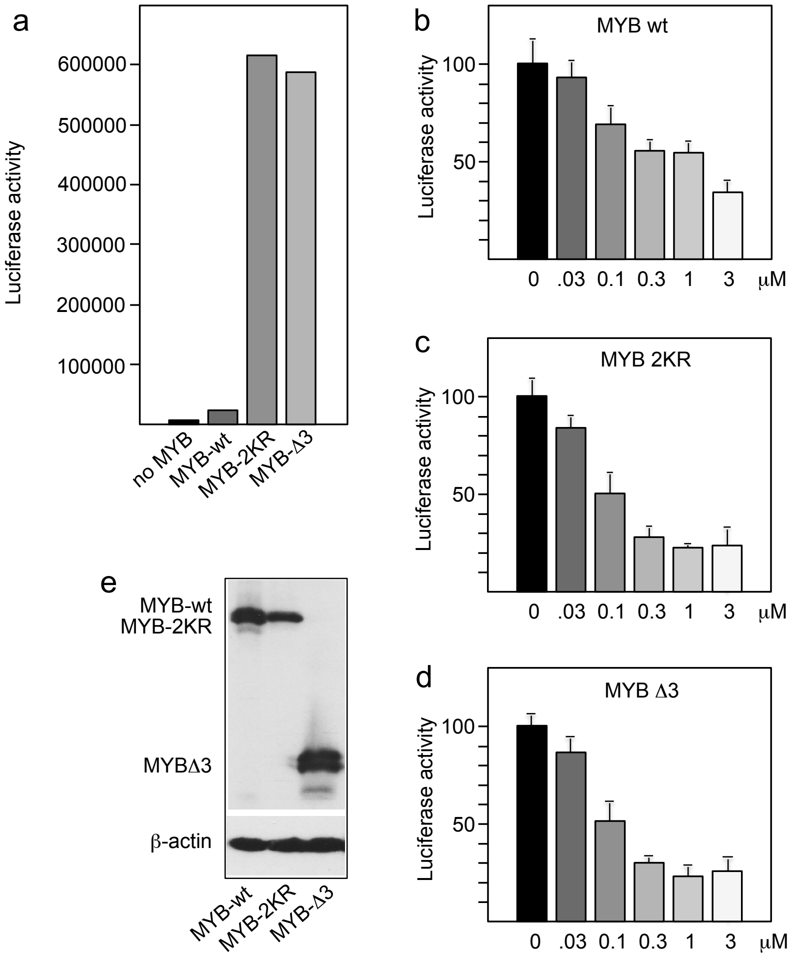

Supplementary Fig.1. Inhibition of wt and mutant Myb by teniposide. Hek293 cells were transiently transfected with the luciferase reporter gene pGL4-5xMRE(GG)-Myc and expression vectors for MYB-wt (b), MYB-2KR (c) or MYB-Δ3 (d). After transfection the cells were pipetted off the plate and equal amounts were replated in a microtiter plate, with 4 replicate wells for each MYB construct and each concentration of teniposide. The cells were incubated over night followed by measuring the luciferase activity. Panel a shows a comparison of the absolute luciferase activities in the absence of teniposide. Panels b to d show the relative luciferase activities in the presence of the indicated concentrations of teniposide. Panel e shows a western blot analysis of the transfected cells using antibodies against MYB and β-actin.

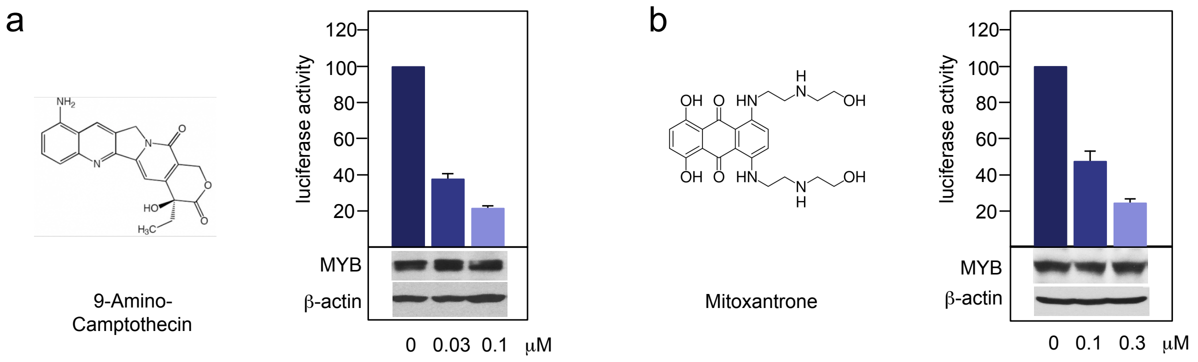

Supplementary Fig. 2. Inhibition of MYB activity by 9-amino-camptothecin and mitoxantrone. The chemical structures of 9-amino-camptothecin (a) and mitoxantrone (**b**) are shown on the left side. The luciferase activity of Hek-Myb-Luc cells after treatment for 12 hours with the respective compounds are shown on the right. The bottom panels show the expression of MYB and β-actin. Columns and standard deviations for luciferase activity are based on three independent experiments with two replicate samples in each case.

Original western blots

A novel cell-based screening assay for small-molecule MYB inhibitors identifies podophyllotoxins teniposide and etoposide as inhibitors of MYB activity

Maria Yusenko, Anke Jakobs and Karl-Heinz Klempnauer

Original western blots for Fig.1b

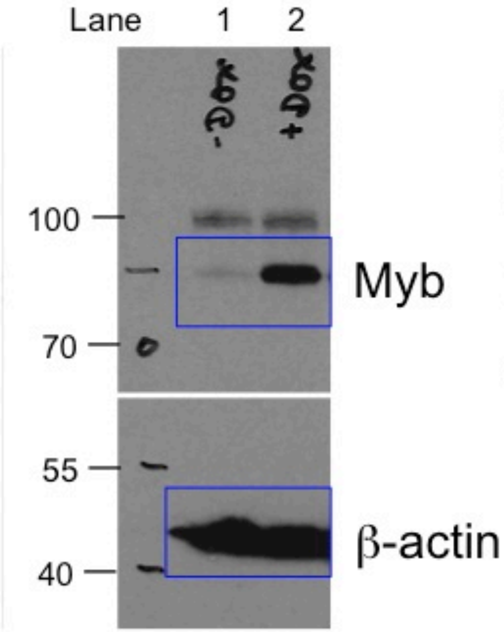

antibodies
top: anti Myb
bottom: anti β-actin

Lanes:
1. – docxycylin
2. + doxycyclin

Original western blots for Fig.1c

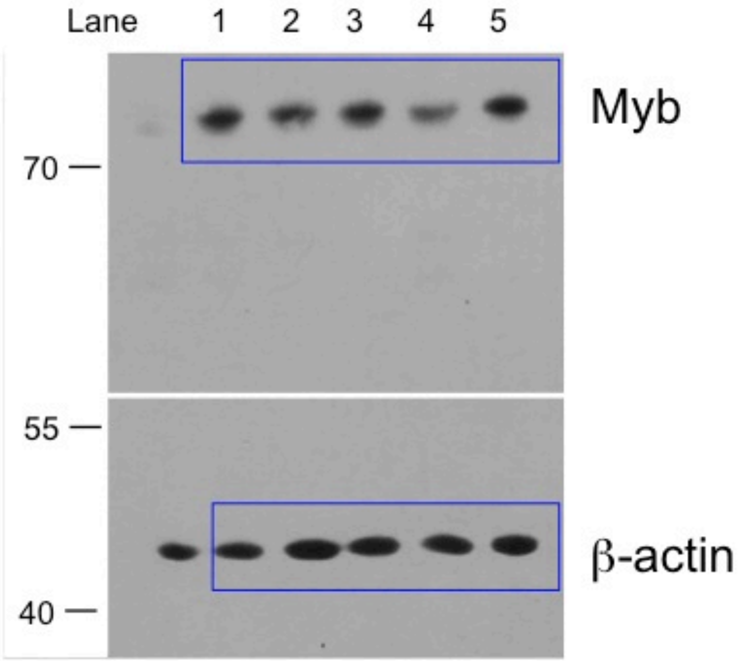

antibodies
top: anti Myb
bottom: anti β-actin

Lanes:

1. – Celastrol
2. + 0,03 μM Cela
3. + 0.1 μM Cela
4. + 0.3 μM Cela
5. + 1 μM Cela

Original western blots for Fig.3b (teniposide)

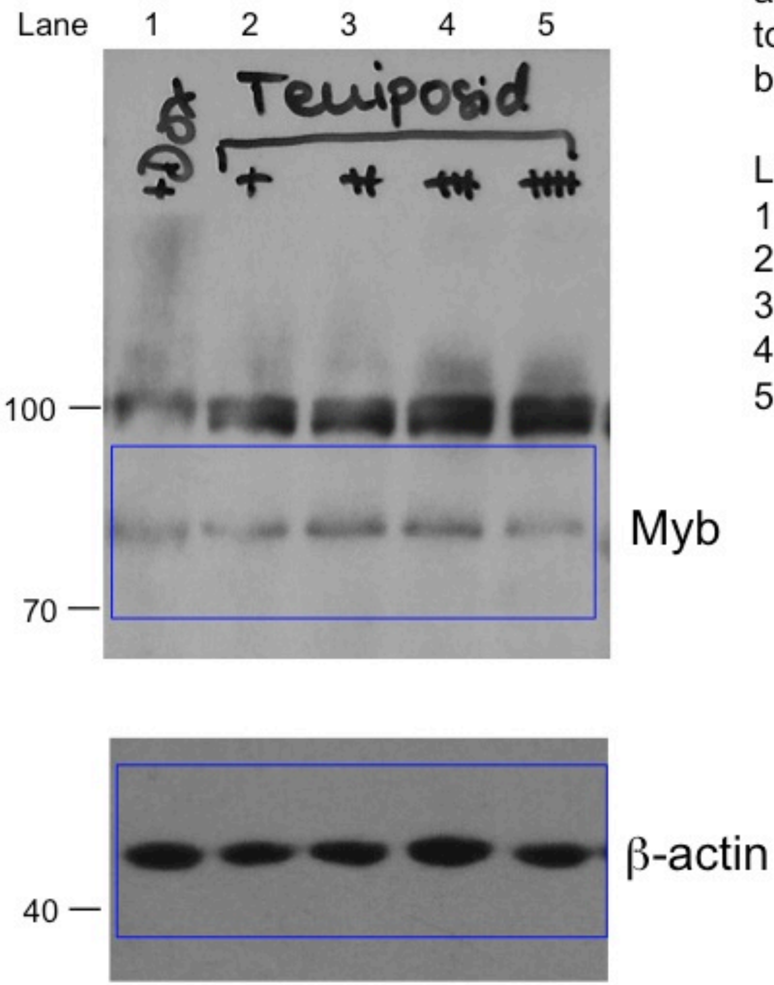

antibodies
top: anti Myb
bottom: anti β-actin

Lanes:

1. – Teniposide
2. + 0,03 μM Teni
3. + 0.1 μM Teni
4. + 0.3 μM Teni
5. + 1 μM Teni

Original western blots for Fig.3b
(toyocamycin)

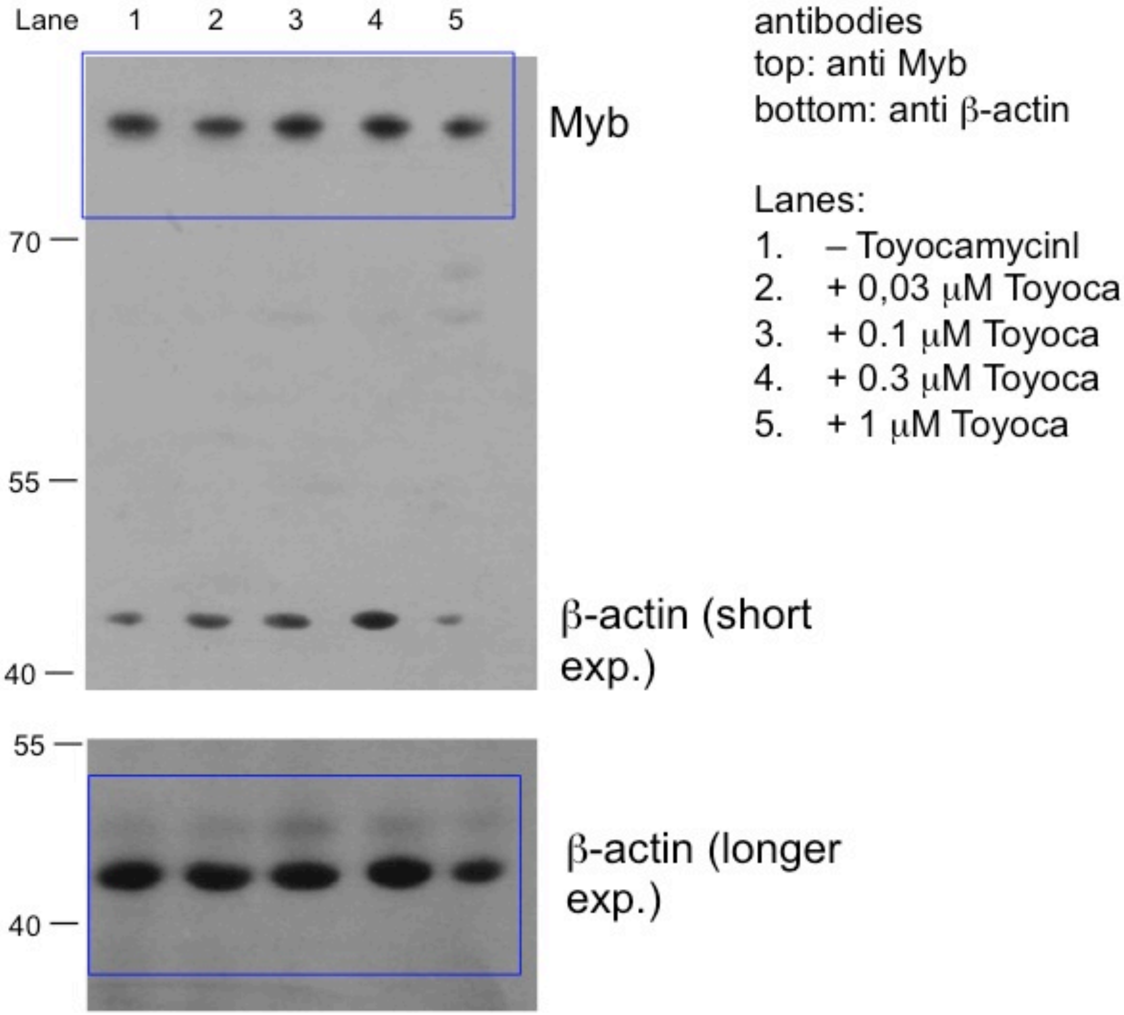

antibodies
top: anti Myb
bottom: anti β-actin

Lanes:
1. – Toyocamycinl
2. + 0,03 μM Toyoca
3. + 0.1 μM Toyoca
4. + 0.3 μM Toyoca
5. + 1 μM Toyoca

Original western blots for Fig.4d

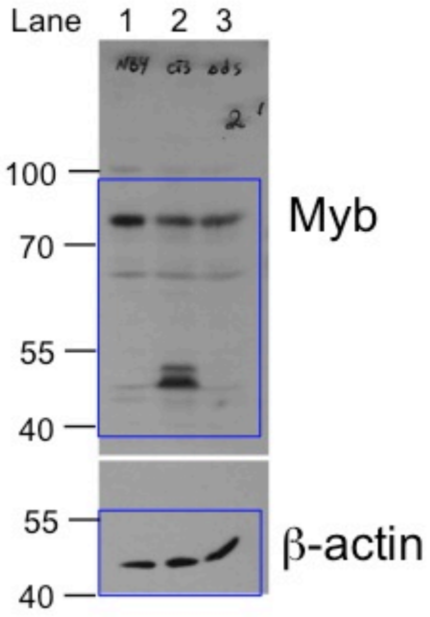

antibodies
top: anti Myb
bottom: anti β-actin

Lanes:

1. NB4 cells
2. NB4MYBΔ3 cells
3. NB4 control cells

Original western blots for Fig.4e

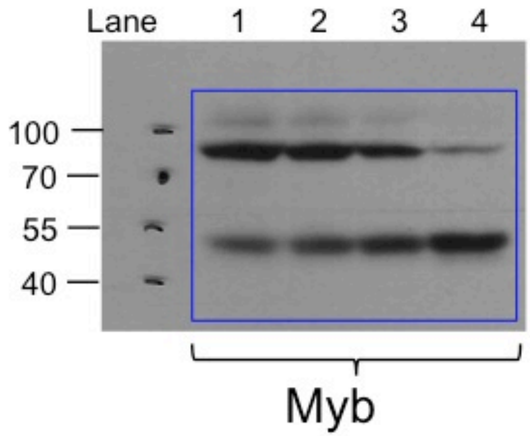

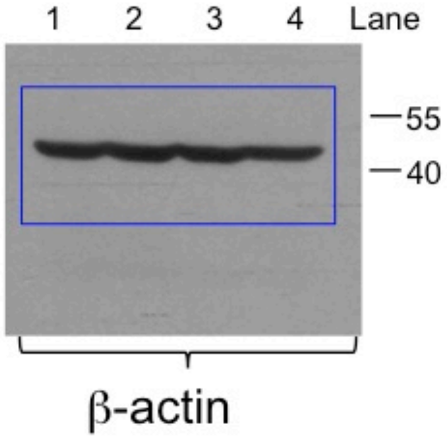

antibodies
left: anti Myb
right: anti β-actin

Lanes:

1. – Teniposide
2. + 30 nM Teniposide
3. + 100 nM Teniposide
4. + 300 nM Teniposide

Original western blots for Fig.5a
(HL60)

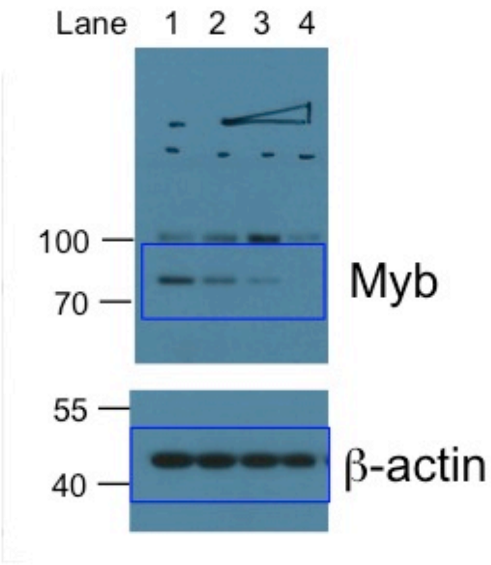

antibodies
top: anti Myb
bottom: anti β-actin

Lanes:
1. – Teniposide
2. + 0,03 μM Teni
3. + 0.1 μM Teni
4. + 0.3 μM Teni

Original western blots for Fig.5a
(NB4)

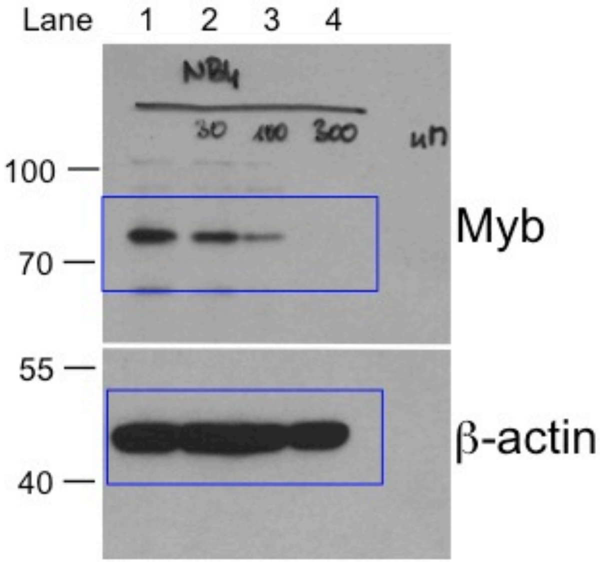

antibodies
top: anti Myb
bottom: anti β-actin

Lanes:
1. – Teniposide
2. + 0,03 μM Teni
3. + 0.1 μM Teni
4. + 0.3 μM Teni

Original western blots for Fig.5a
(U937)

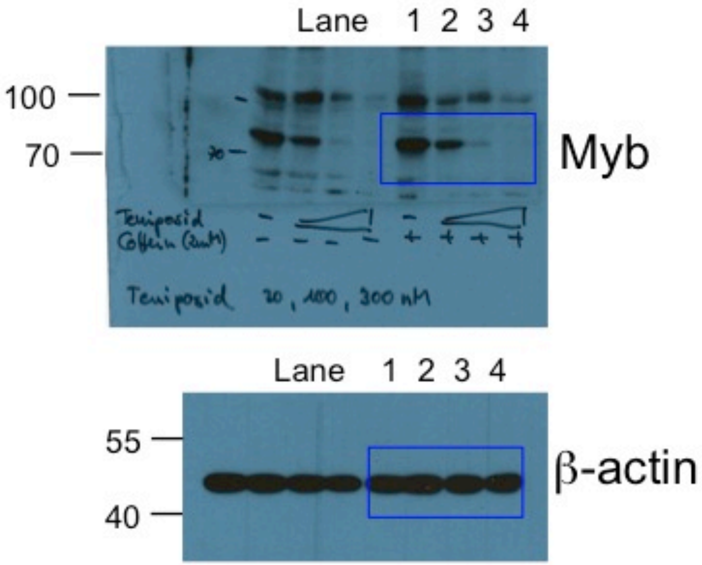

antibodies
top: anti Myb
bottom: anti β-actin

Lanes:

1. – Teniposide
2. + 0,03 μM Teni
3. + 0.1 μM Teni
4. + 0.3 μM Teni

Original northern blots for Fig.5b

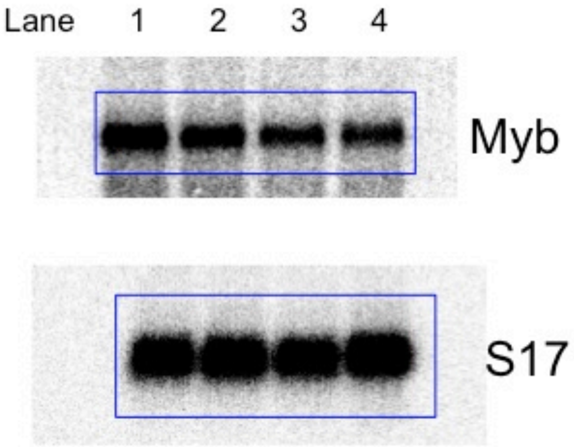

Northern blot probes
top: Myb
bottom: S17

Lanes:
1. – Teniposide
2. + 0,03 μM Teni
3. + 0.1 μM Teni
4. + 0.3 μM Teni

Original western blots for Fig.5c

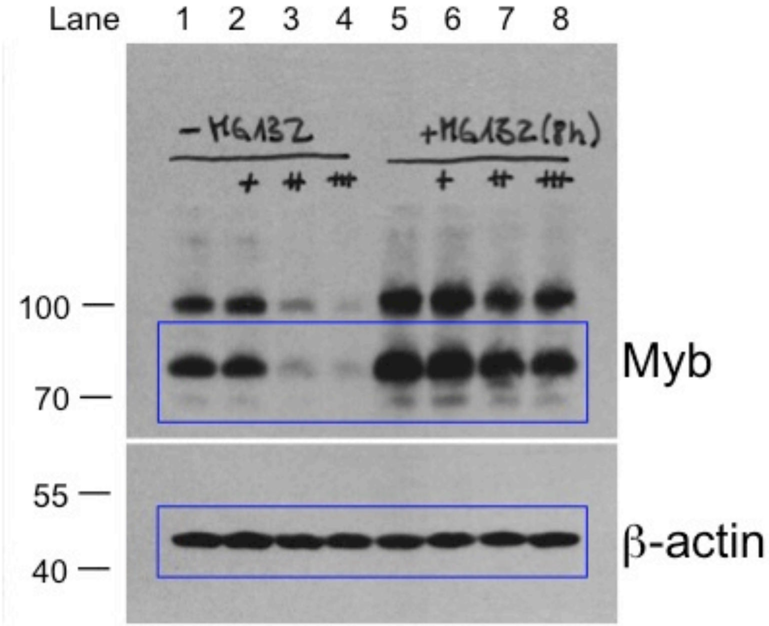

antibodies
top: anti Myb
bottom: anti β-actin

Lanes:
1. – Teniposide
2. + 0,03 μM Teni
3. + 0.1 μM Teni
4. + 0.3 μM Teni
5. - Teniposide, + MG132
6. + 0,03 μM Teni, + MG132
7. + 0.1 μM Teni, + MG132
8. + 0.3 μM Teni, + MG132

Original northern blots for Fig.5d

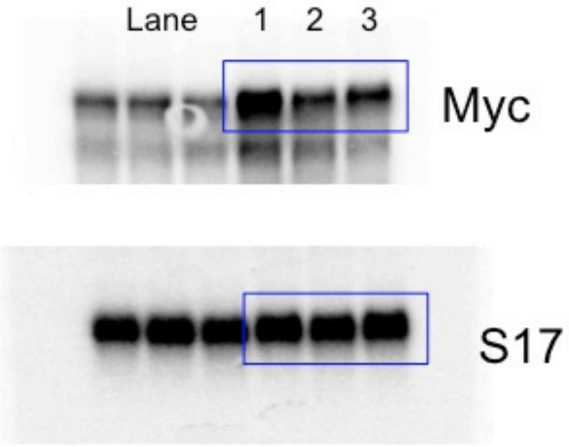

Northern blot probes
top: Myc
bottom: S17

Lanes:

1. – Teniposide
2. + 0,03 μM Teni
3. + 0.1 μM Teni
4. + 0.3 μM Teni

Original western blots for Fig.5d

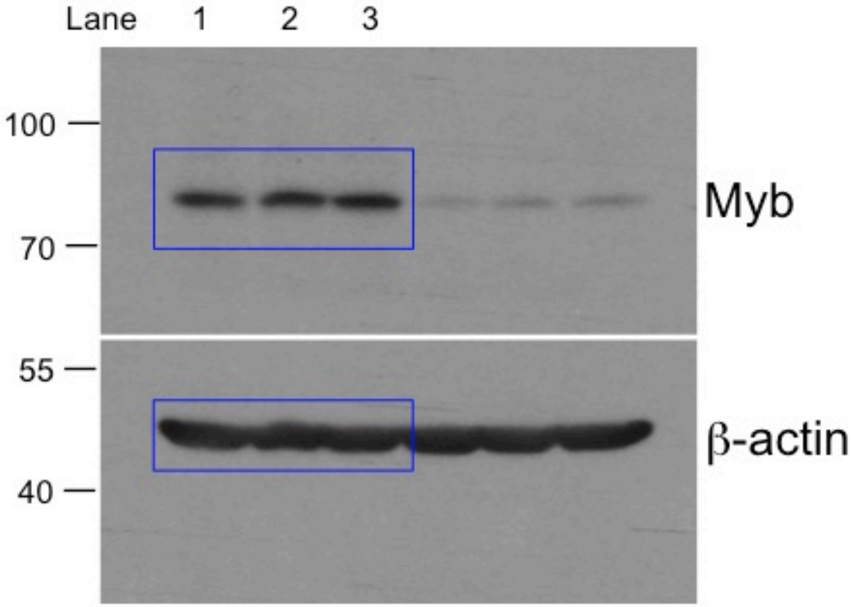

antibodies
top: anti Myb
bottom: anti β-actin

Lanes:
1. – Teniposide
2. + 100 nM Teni
3. + 300 nM Teni

Original western blots for Fig. 5f

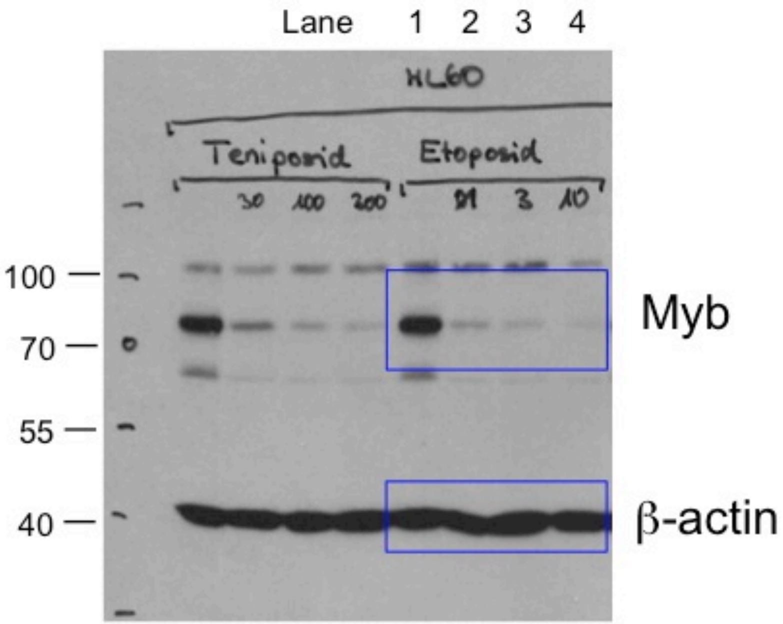

antibodies
top: anti Myb
bottom: anti β-actin

Lanes:
1. – Etoposide
2. + 1 μM Etoposide
3. + 3 μM Etoposide
4. + 10 μM Etoposide

Original western blots for Fig.6b

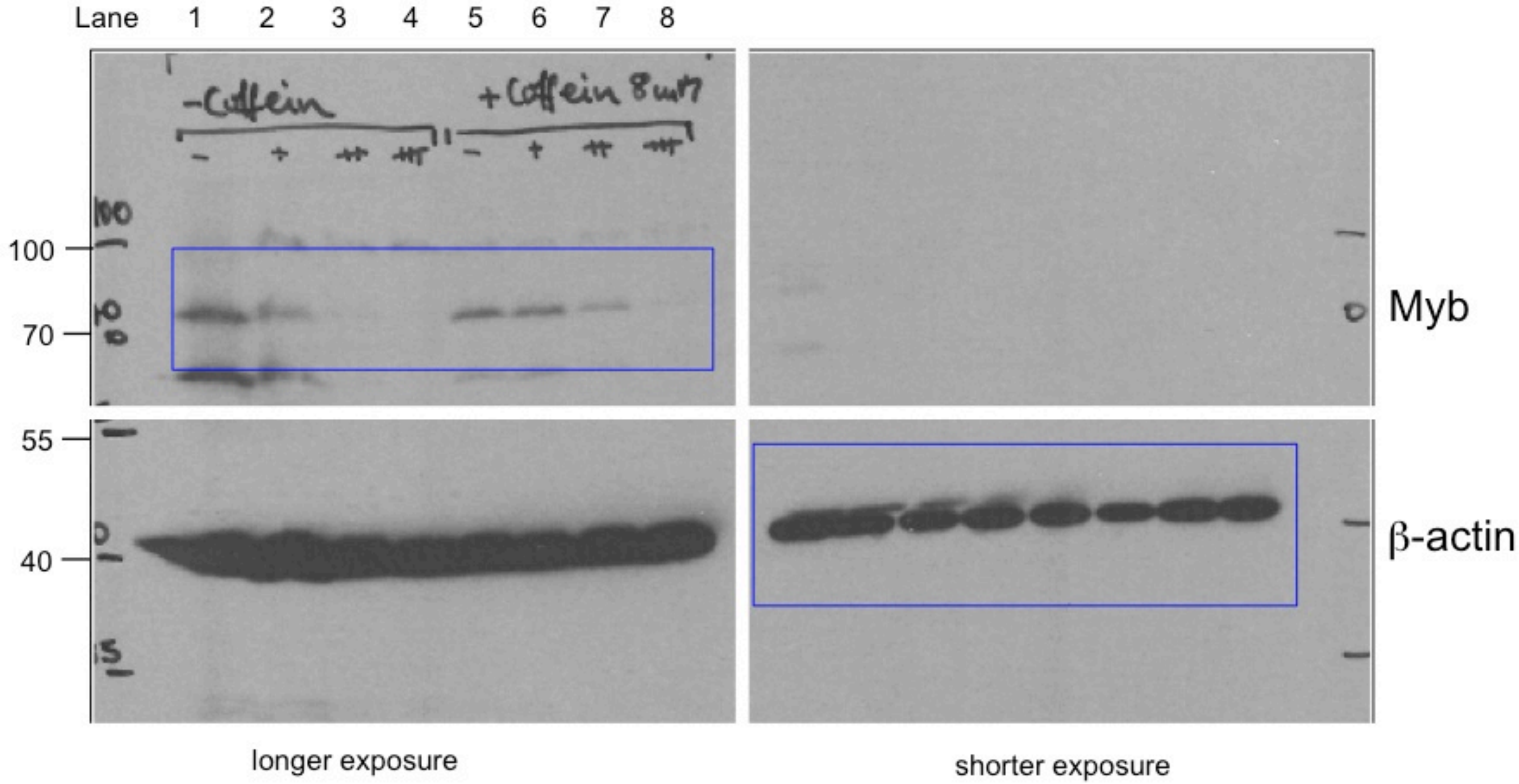

antibodies
top: anti Myb
bottom: anti β-actin

Lanes:

1. – Teniposide
2. + 30 nM Teni
3. + 100 nM Teni
4. + 300 nM Teni
5. – Teniposide
6. + 30 nM Teni, + caffeine
7. + 100 nM Teni, + caffeine
8. + 300 nM Teni, + caffeine

Original western blots for Fig.6c
(Myb and β-actin)

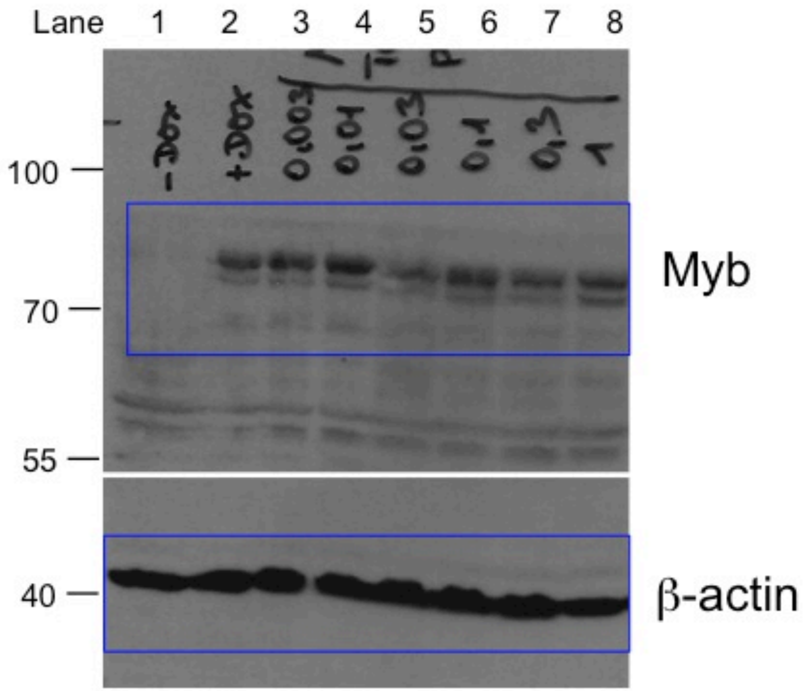

antibodies
top: anti Myb
bottom: anti β-actin

Lanes:

1. - dox, - Teniposide
2. + dox, - Teniposide
3. + dox, +0,003 μM Teni
4. + dox, + 0.01 μM Teni
5. + dox, + 0.03 μM Teni
6. + dox, + 0.1 μM Teni
7. + dox, + 0.3 μM Teni
8. + dox, + 1 μM Teni

Original western blots for Fig.6c
(γ-H2AX)

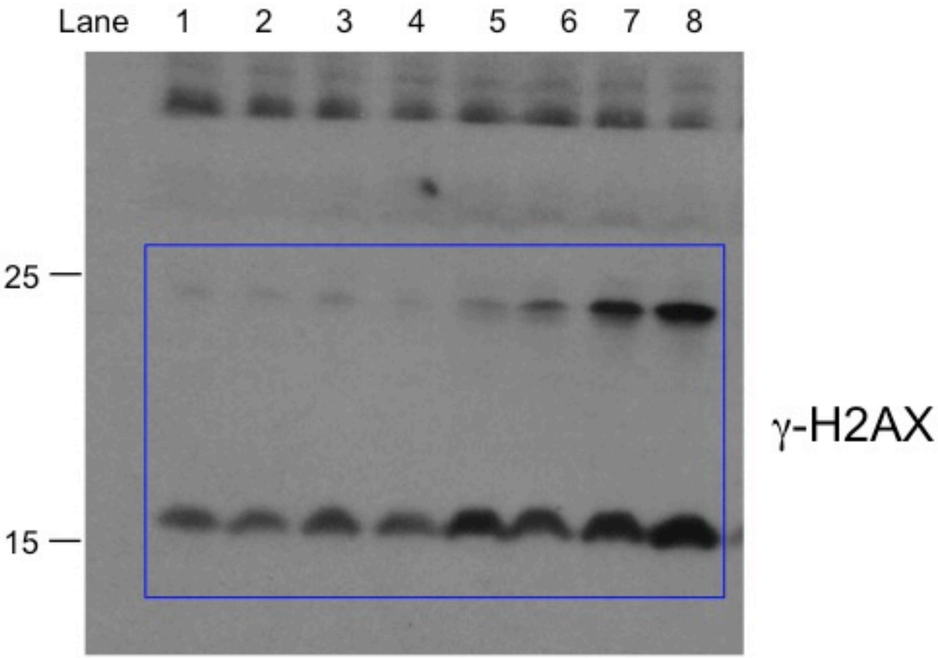

antibodies
Anti γ-H2Ax

Lanes:

1. - dox, - Teniposide
2. + dox, - Teniposide
3. + dox, +0,003 μM Teni
4. + dox, + 0.01 μM Teni
5. + dox, + 0.03 μM Teni
6. + dox, + 0.1 μM Teni
7. + dox, + 0.3 μM Teni
8. + dox, + 1 μM Teni

Original western blots for Fig.6c
(p53)

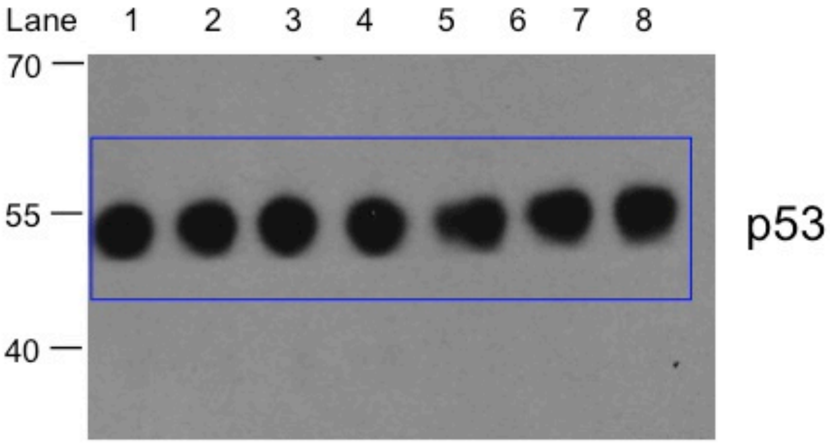

antibodies
anti p53

Lanes:
1. - dox, - Teniposide
2. + dox, - Teniposide
3. + dox, +0,003 μM Teni
4. + dox, + 0.01 μM Teni
5. + dox, + 0.03 μM Teni
6. + dox, + 0.1 μM Teni
7. + dox, + 0.3 μM Teni
8. + dox, + 1 μM Teni

Original western blots for Fig. 6d

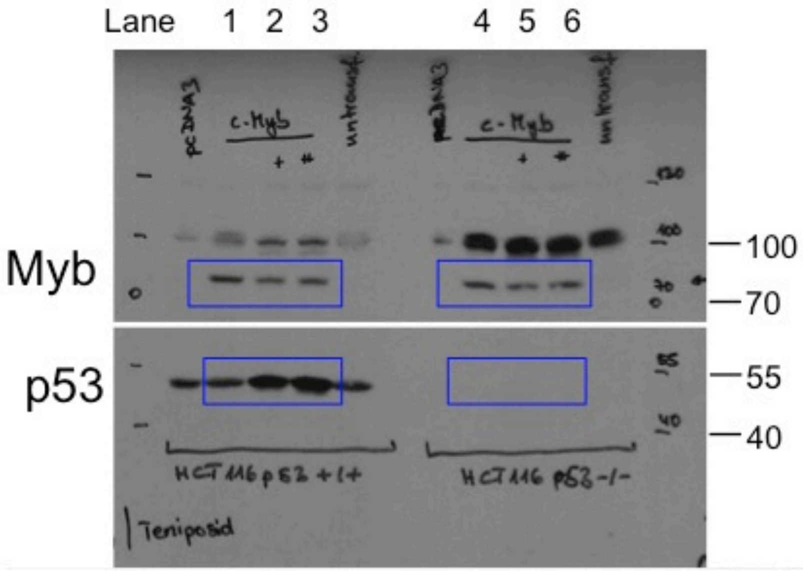

antibodies
top: anti Myb
bottom: anti p53

Lanes:

1. - Teniposide, HCT116 wt
2. + 1 μM Teni, HCT116 wt
3. + 3 μM Teni, HCT116 wt
4. - Teniposide, p53 knock-out
5. + 1 μM Teni, p53 knock-out
6. + 3 μM Teni, p53 knock-out

Original western blots for supplementary Fig.1 (Myb)

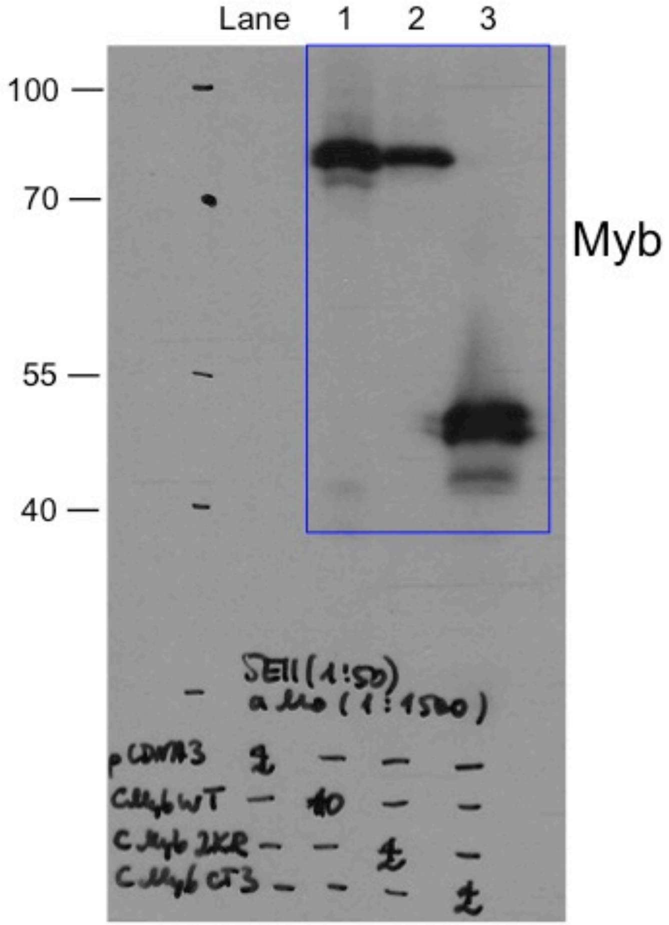

Antibodies:
anti Myb

Lanes:

1. Myb-wt cells
2. Myb-2KR cells
3. Myb-Δ3 cells

Original western blots for supplementary Fig.1
(β-actin)

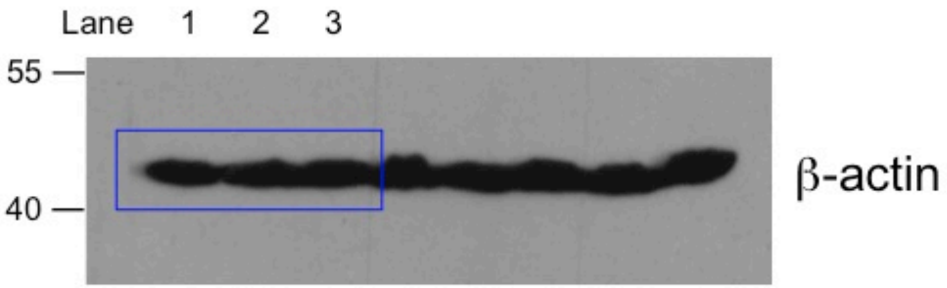

Antibodies:
anti β-actin

Lanes:
1. Myb-wt cells
2. Myb-2KR cells
3. Myb-Δ3 cells

Original western blots for supplementary Fig.2

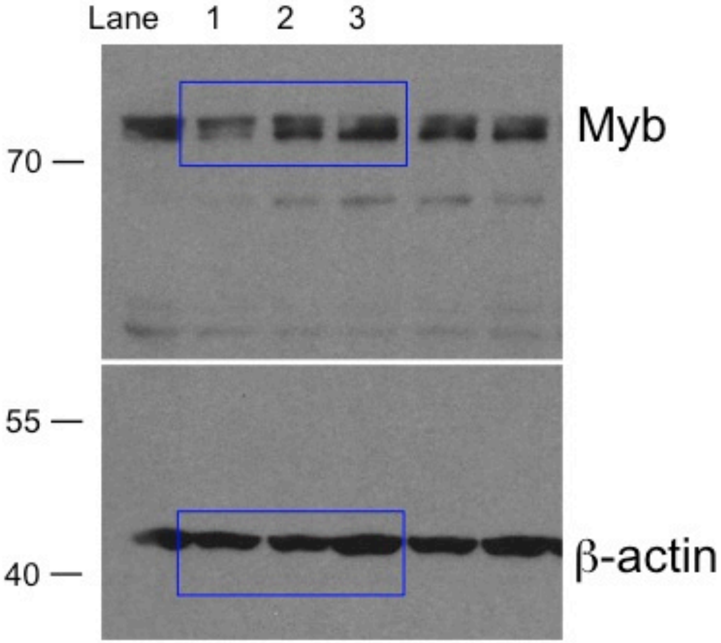

Antibodies:
top: anti Myb
bottom: anti β-actin

Lanes:
1. - Camptothecin
2. + 0.03 μM Camptothecin
3. + 0.1 μM Camptothecin

Original western blots for supplementary Fig.2b

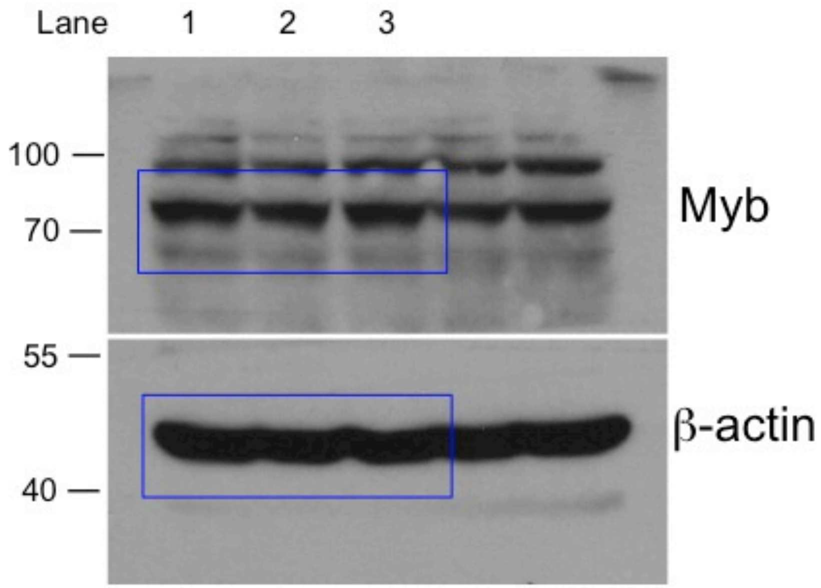

Antibodies:
top: anti Myb
bottom: anti β-actin

Lanes:
1. - Mitoxantrone
2. + 0.1 μM Mitoxantrone
3. + 0.3 μM Mitoxantrone
